# Editorial: Bioinformatics of Genome Regulation, Volume I

**DOI:** 10.3389/fgene.2021.803273

**Published:** 2021-12-06

**Authors:** Yuriy L. Orlov, Tatiana V. Tatarinova, Nina Y. Oparina, Elvira R. Galieva, Ancha V. Baranova

**Affiliations:** ^1^ Institute of Digital Medicine, I.M.Sechenov First Moscow State Medical University (Sechenov University), Moscow, Russia; ^2^ Agrarian and Technological Institute, Peoples’ Friendship University of Russia (RUDN University), Moscow, Russia; ^3^ Life Sciences Department, Novosibirsk State University, Novosibirsk, Russia; ^4^ Institute of Cytology and Genetics SB RAS, Novosibirsk, Russia; ^5^ Natural Science Division, La Verne University, La Verne, CA, United States; ^6^ Institute of Medicine, University of Gothenburg, Göteborg, Sweden; ^7^ School of Systems Biology, George Mason University, Fairfax, VA, United States; ^8^ Research Centre for Medical Genetics, Moscow, Russia

**Keywords:** bioinformatics, computational genomics, gene expression, gene expression regulation, model organisms, genetics

This “*Bioinformatics of Genome Regulation*” issue presents the studies in the field of bioinformatics of gene expression regulation. The materials were initially discussed in part at BGRS\SB-2020 (Bioinformatics of Genome Regulation and Structure\Systems Biology) multi-conference (https://bgrssb.icgbio.ru/2020) in Novosibirsk, Russia. The BGRS conference series is organized by the Institute of Cytology and Genetics SB RAS every other year since 1998. This issue consists of two parts—Volume I (https://www.frontiersin.org/research-topics/14266/bioinformatics-of-genome-regulation-volume-i) and Volume II (https://www.frontiersin.org/research-topics/17947/bioinformatics-of-genome-regulation-volume-ii). It continues the tradition of Research Topics at Frontiers in Genetics journal (https://www.frontiersin.org/research-topics/8383/bioinformatics-of-genome-regulation-and-systems-biology), which presents the works discussed at the BGRS-2018 meeting (Orlov et al., 2016; Tatarinova et al., 2019). Previous journal covering BGRS\SB conference were presented in the Journal of Bioinformatics and Computational Biology in 2012 (Orlov et al., 2015; Orlov et al., 2019a) and other platforms (Chen et al., 2017; Baranova et al., 2019; Orlov, 2019; Orlov, 2019b; Orlov et al., 2021a). Starting in 2018, actual research works on the mechanisms of gene expression regulation are presented in Frontiers in Genetics being extended as Volume II.

In Volume I of this Research Topic a total of 19 papers were arranged by two main areas—biomedical bioinformatics for human health and plant model studies. Biomedical papers start from bioinformatics applications to various cancers, including hepatocellular carcinoma, melanoma, brain tumors, prostate cancers, and paraganglioma.

Li et al. described a bioinformatics pipeline to reveal critical genes associated with hepatocellular carcinoma. The authors analyzed differentially expressed genes, followed by the Reconstruction of the protein-protein interaction (PPI) network. Eight hub genes significantly upregulated in carcinoma samples were highlighted and validated using GEPIA (Gene Expression Profiling Interactive Analysis) and Oncomine databases.

Fedorova et al. studied *NETO2* gene (neuropilin and tolloid-like 2) upregulation in diverse tumors, including ones originating in breast, prostate, and colorectal tissues. In addition, the authors evaluated *NETO2* functions in a short-lived fish model *Nothobranchius furzeri*.

Liu et al. consider how cutaneous melanoma involves ERBB tyrosine kinase family members (ERBB receptor family) in its progression.

Wang et al. studied immune surveillance within the microenvironment in glioma. The authors highlighted long non-coding RNA (lncRNA) as the key in glioma progression.

Pudova et al. showed how the gene expression landscapes change during the progression of prostate cancer to its advanced stages. They described relevant networks and pathways pertinent to early recurrence. Rare neuroendocrine tumors were studied in Snezhkina et al. for the frequencies of the mutations within susceptibility genes such as *SDHx*. This work aids in understanding the immunochemistry analysis of *SDHx* genes in carotid paragangliomas reported earlier (Snezhkina et al., 2020).

Mukushkina et al. applied bioinformatics tools to study how miRNAs interact with other genes’ products form atherosclerotic plaques.

Frontiers in Genetics’ publications on human disease biomarkers are continued by the Research Topic “*High-throughput sequencing-based investigation of chronic disease markers and mechanisms*” (https://www.frontiersin.org/research-topics/21036/high-throughput-sequencing-based-investigation-of-chronic-disease-markers-and-mechanisms). Thus, Shi et al. (2021) studied molecular mechanisms related to alternative polyadenylation in gastric cancer; Chang et al. (2021) analyzed platinum-drug resistance mutations in advanced non-small cell lung cancer.

In this “*Bioinformatics of Genome Regulation*” Topic Yao et al. have reported the results of a search for molecular markers associated with complications of systemic lupus erythematosus.

Skuratovskaia et al. described the mechanisms regulating carbohydrate metabolism in Type 2 diabetes mellitus using the bioinformatics framework.

The approaches for analysis of gene expression regulation in human diseases from the population genetics point of view were highlighted in the Research Topic “*Association Between Individuals’ Genomic Ancestry and Variation in Disease Susceptibility*” at Frontiers in Genetics (https://www.frontiersin.org/research-topics/15891/association-between-individuals-genomic-ancestry-and-variation-in-disease-susceptibility). Kamenova et al. (2021) analyzed gene expression regulation by miRNA in Parkinson’s disease. Zinchenko et al. (2021) studied rare hereditary diseases in Russia. Ramensky et al. (2021) discussed targeted sequencing of a set of clinically important genes associated with cardiovascular diseases.

Gozman et al. (2021) raised important actual problem of the role of genetic variance in disease severity in COVID-19 Patients. This problem is continuing to be actively discussed (Lu et al., 2021).

The following two articles in the Volume I highlight the mechanisms of epigenetic control revealed by gene network reconstruction in animal models. Shen et al.investigated the changes of DNA methylation and hydroxymethylation in mouse genome during puberty an emphasis on the activation of by hypothalamic gonadotropin-releasing hormone pathway.

Adonin et al. applied the methods of gene networks Reconstruction to a fascinating model organism—sea urchin *Strongylocentrotus purpuratus*.

The next group of articles in this Research Topic performed gene expression analysis in plants. This science field was presented at the bioinformatics conference series in Novosibirsk (Orlov et al., 2019b). In particular, Marla et al. performed the plant genome assembly from short sequencing reads of Pigeonpea (*Cajanus cajan*). Chakraborty et al. annotated miRNA functions in various millet species. Samarina et al. studied cold-resistance genes in the tea plant *Camellia sinensis*. Cold and drought stresses cause osmotic changes in the cells of the tea plant (Samarina et al., 2020). This study identified 45 stress-inducible candidate genes associated with cold and drought responses using homologous detection in related plant species. The gene network analysis revealed upregulated expression in the *ICE1*-related cluster of *bHLH* factors. Pavlinova et al. presents another application of network analysis in plant science; dynamical modeling of the core gene network controlling the transition to flowering in *Pisum sativum* was performed to extend previously developed non-linear regression models of the flowering in wild chickpea (Kozlov et al., 2019).

Finally, a set of novel computational techniques was developed for deciphering gene expression regulation in cells. Arega et al. presented a novel tool for 3D genomics modeling of long-range chromatin interactions, the ChIAMM algorithm, which utilizes ChIA-PET (Chromatin Interaction Analysis by Paired-End Tags sequencing) to estimate amounts of chromosome contacts (loops) mediated by a given transcription factor. The same authors’ group has also described a 3D genome structure in cervical cancer cells (Adeel et al., 2021). The topic of genome architecture prediction based on 3D interaction maps in cell nuclei was further advanced by Belokopytova and Fishman. The authors reviewed high-throughput genome-wide chromatin profiling and chromosome contacts mapping using chromosome conformation capture techniques (Hi-C and ChIA-PET). The Research Topic “*The Role of High-Order Chromatin Organization in Gene Regulation*” (https://www.frontiersin.org/research-topics/18088/the-role-of-high-order-chromatin7-organization-in-gene-regulation ) has been put together at Frontiers in Genetics by Drs. Fishman and Pindyurin.

In their brief report, Glyakina and Galzitskaya discuss bioinformatics modeling of actin molecules. Biziukova et al. utilized Machine Learning–based analysis of the scientific texts in HIV treatment to systematize information on small molecules, proteins, and genes related to the disease.

Overall, we are proud of the continuing Research Topic at Frontiers in Genetics we collated. We hope that you will find this paper collection a stimulating reading and consider coming to the next BGRS\SB conferences in Novosibirsk, Russia (https://bgrssb.icgbio.ru/2022/), and read the next continuing Research Topics in Frontiers (https://www.frontiersin.org/research-topics/21036/high-throughput-sequencing-based-investigation-of-chronic-disease-markers-and-mechanisms).
